# Application of a Protocol to Assess Camel Welfare: Scoring System of Collected Measures, Aggregated Assessment Indices, and Criteria to Classify a Pen

**DOI:** 10.3390/ani11020494

**Published:** 2021-02-13

**Authors:** Laura Menchetti, Martina Zappaterra, Leonardo Nanni Costa, Barbara Padalino

**Affiliations:** Department of Agricultural and Food Sciences, University of Bologna, Viale Fanin 46, I-40127 Bologna, Italy; laura.menchetti@unibo.it (L.M.); martina.zappaterra2@unibo.it (M.Z.); leonardo.nannicosta@unibo.it (L.N.C.)

**Keywords:** camel, welfare, overall assessment, animal-based indicators, livestock market

## Abstract

**Simple Summary:**

During the last few decades, several protocols have been developed for assessing the on-farm welfare of several animal species. However, a protocol for camels has only recently been proposed. This study, for the first time, applied this assessment protocol and developed a model to compound overall welfare indices and classify pens according to their welfare level. The welfare measures were collected in 76 pens of a camel market in Qatar, scored, and then aggregated to obtain overall welfare indices. Thirst Index, Body Condition Score (BCS), disease and physical injuries, feeding and watering management, presence of a shelter, and cleanliness of bedding were the measures that strongly affected the classification of the pens. The model seemed to be able to identify the major welfare concerns of camels kept at the market and to suggest corrective actions. Further studies are needed to implement the proposed model, but it may be the first step towards the definition of welfare standards for camels.

**Abstract:**

This study aimed to apply a protocol for assessing camel welfare, to develop a scoring system for the welfare measures, to produce overall assessment indices, and to classify the animal units (i.e., pens) according to their welfare level. A total of 105 measures were collected at Herd level from 76 pens at a market in Qatar. The pens held 528 camels, 132 of which were evaluated at a deeper level (i.e., Animal level). Out of the 105 measures, 71 were selected, scored, and aggregated to reach a Total Welfare Index (TWI) for each pen. The TWI ranged from 46.2 to 69.8. The Good Feeding index, including measures related to prolonged thirst and prolonged hunger, was the most critical (*p* < 0.001), while the Good Health index, including measures related to the absence of injuries, disease and pain, was the less problematic (*p* < 0.001). However, most of the pens were classified as “unsatisfactory” (61.8%) and none as “excellent”. Body Condition Score (BCS), Thirst Index, disease and physical injuries, presence of a shelter, and cleanliness of bedding were the measures which influenced the pens’ classification the most (*p* < 0.05). The proposed model seems useful in the identification of camel welfare issues. Further applications, as well as the involvement of many scientists and stakeholders, are needed to refine and validate the protocol and its indices.

## 1. Introduction

Animal welfare has become very important for public opinion, with recognized effects on public health and food production sustainability. During the last 40 years, the results of scientific studies on animal welfare have led to increased public concern on the quality of life of animals kept in intensive systems [1,2]. This growing awareness highlighted the need for assessing the actual welfare conditions of animals to provide information on the influences of their living conditions [3]. Animal welfare measures how an animal is coping with the conditions in which it lives [4] and is a scientific concept describing a potentially measurable state of a living animal [1]. Animal welfare is a complex concept embracing several aspects (i.e., absence of suffering, high levels of biological functioning and “positive emotions”) [2,5]. Therefore, assessing welfare requires a multidimensional approach [6] with measures aimed to determine both physical and mental states of the animals [7].

The first project attempting to create a protocol for assessing animal welfare through a multidimensional approach was named Welfare Quality^®^ [8]. This European project set up the Welfare Quality^®^ protocol, which defined four welfare principles (Good Feeding, Good Housing, Good Health and Appropriate Behaviour), originating from the Five Freedoms [9,10], and different criteria for each principle [11]. The Welfare Quality^®^ protocol comprised animal-, resource- and management-based measures. While animal-based measures evaluate animals’ physical and psychological health status and are indicators of welfare-related outcomes [7,12], resource- and management-based measures are mainly related to the housing environment and could indicate factors with the potential to cause poor welfare outcomes, i.e., hazard [7,12,13]. At a later time, the European Commission then funded the Animal Welfare Indicators (AWIN) research project covering species not yet considered in Welfare Quality^®^ [14,15,16]. Both projects aimed to develop assessment protocols that can be easily put into practice in on-farm conditions. However, camels were not considered in both projects.

The aforementioned protocols still face great challenges in their practical implementation. The final aggregation of the measures into an overall welfare index remains complicated [17,18]. In the literature, different strategies for the aggregation of measures have been proposed for compounding an overall welfare assessment (reviewed in Botreau et al., [18]). A simpler strategy is the non-formal aggregation of measured data, such as a formal opinion to a group of experts. Another simple strategy is the comparison of the collected measures with minimal requirements (i.e., check-lists of critical points). More standardized methods to aggregate the measures include a sum of ranks (the measures obtained in a farm have the same importance and are aggregated by summing them) and a weighted sum of scores (i.e., scores obtained on several measures by a farm are given different weights and summed). The main criticisms levelled at these methods are the following: they are complicated, and often lacking in the identification of welfare risk factors or in the suggestion of corrective actions [19,20]. A user-friendly methodology and interpretation of the data are important in a welfare assessment protocol.

Welfare assessment protocols are the basic tools to influence new legislation. They are indeed crucial for developing certification systems, comparing welfare conditions between different farms, and indicating preventive, mitigating and corrective actions to farmers [3,18,20]. Despite the efforts to optimize welfare assessment protocols in many animal species, research advances and specific welfare laws for camel farming are still limited [21]. To date, the only tool for assessing the welfare of camels was developed by Padalino and Menchetti [22]. This protocol includes a combination of animal-, resource- and management-based measures assessed at three levels of investigation: Caretaker level (using a face-to-face interview), Herd level (checking the herd and the pen facilities), and Animal level (inspecting individual camel behaviour and health status). The proposed measures were presented for each welfare principle according to the Welfare Quality^®^ and AWIN methods [13,14]. However, the protocol for assessing camel welfare [22] has not been applied yet, and an aggregation method of the measures to produce an overall welfare assessment has not been proposed.

The aims of this study were, consequently, to apply the protocol for assessing camel welfare proposed by Padalino and Menchetti [22] at the camel market in Doha, and to classify the tested pens according to their welfare level. To achieve the before mentioned aims, the following steps were proposed: (i) developing a scoring system for the measures included in the protocol, (ii) determining an aggregate index for each welfare principle and level of investigation, (iii) producing an overall assessment index, and (iv) proposing criteria for the classification of animal units (i.e., pen where camels are kept).

## 2. Materials and Methods

The research project was run with the permission of the Department for Agriculture Affairs and Fisheries of the Ministry of Municipality and Environment of the State of Qatar. The study involved no invasive sampling methods and all data collection was performed without disturbing the animals. Oral owners’ consents were received before the assessment.

### 2.1. Animals, Housing, and Caretakers

This study was carried out at the permanent camel market in Doha (Qatar) from the 11 to the 18 of September 2019, with an average temperature and humidity of 42.3 °C (range: 36.6–0.3 °C) and 32.2% (range: 15.7–54.4%), respectively. The Temperature Humidity Index (THI) ranged from 84.1 to 102.3 (mean ± standard deviation = 89.1 ± 2.9). At the market, there were 92 pens of different sizes, ranging from 26 m^2^ to 255 m^2^ (median = 167 m^2^). Some pens had some areas that were not available to the camels, as various materials such as broken furniture, field kitchens, and camp beds were stored inside the pens. During the study, 16 pens were empty, so only 76 pens, where at least one camel was kept, were included in the data collection. In these pens, there was a total of 528 camels of different genders, ages, and geographical origins (i.e., Qatar, Sudan, Oman, Saudi Arabia, United Arab Emirates, Kuwait, Pakistan, and Somalia). The camels were kept at the market for different purposes, namely meat, milk, breeding or racing, and some were permanently kept (e.g., milk and breeding purposes) while others were kept only for shorter periods and sold for slaughter or live trade. The camels were owned by different people. Each owner employed one or more caretakers to manage their camels, some of which were housed inside the pen of the camels. The caretakers were all males, and mainly from Sudan (91.7%).

### 2.2. Protocol

The welfare protocol applied in the present study was described in detail by Padalino and Menchetti [22]. Table 1 summarises the measures according to welfare principles and levels of investigation suggested by the protocol.

Five people with a solid scientific background in camel behaviour, health, and welfare (i.e., assessors) carried out the data collection in the morning, from 7:00 a.m. to 11:00 a.m. The assessors had previously been trained on the protocol and its welfare measures. Initially, a meeting with the camels’ owners and caretakers was organised; a native Arabic speaker approached them and mediated the meeting, during which the objectives and methods of the welfare protocol were explained and the animal ethics approval was gained by oral confirmation. During the meeting, each caretaker was also asked to provide information concerning the possible aggressive behaviour of particular camels in order to keep assessors, workers, and camels safe during the visit. All the procedures were conducted without interfering with the work routine of caretakers and avoiding disturbance to the animals.

**Figure 1:**
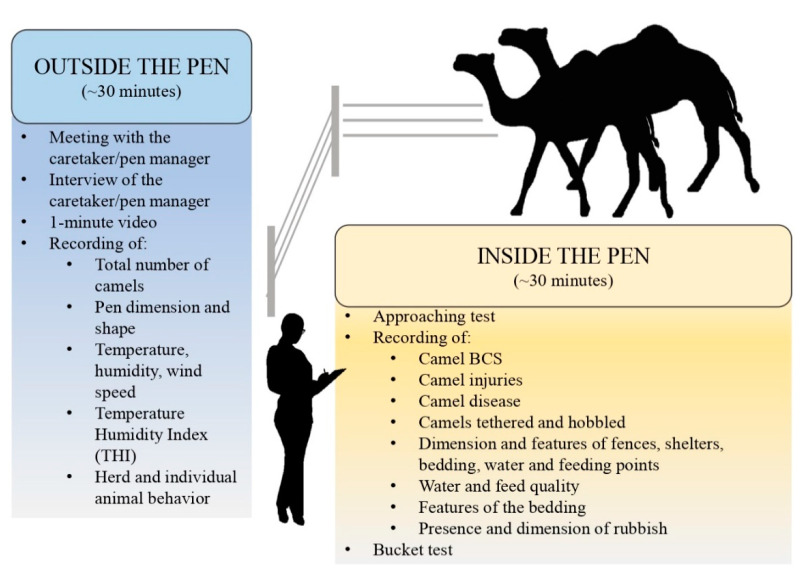
Camel welfare assessment: flow of steps of the protocol proposed by Padalino and Menchetti [22]. BCS = Body Condition Score.

**Figure 2:**
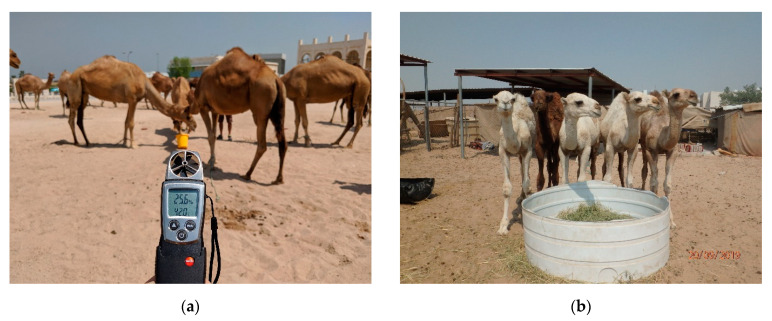

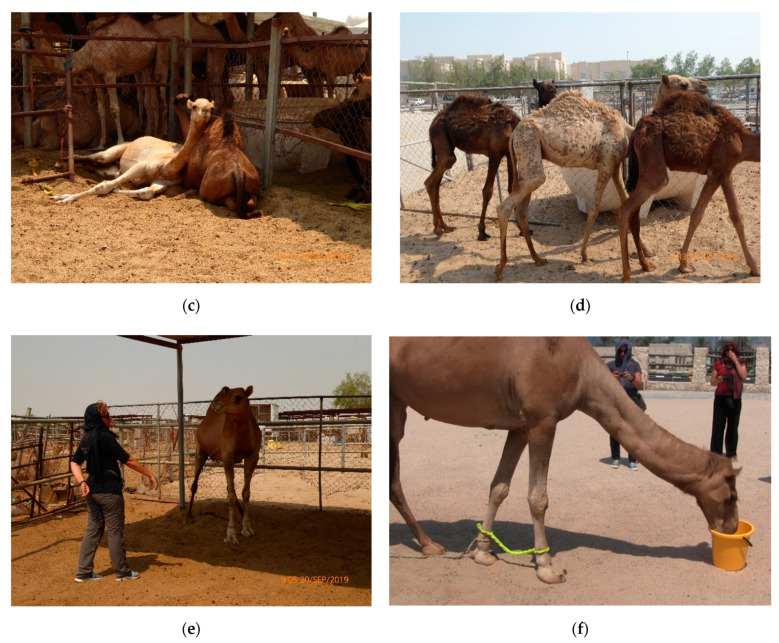
Camel welfare assessment: collection of some measures included in the camel welfare assessment protocol proposed by Padalino and Menchetti [22] at the Doha market (Qatar): (**a**) environmental parameters measured using a weather station; (**b**) placement of feeding point (in the sun), feed availability and quality; (**c**) presence of social interactions; (**d**) presence of disease (skin disorders); (**e**) approaching test to a camel showing hobbles’ scars; (**f**) bucket test to a tethered camel wearing hobbles.

The assessment started with the Caretaker level, consisting of a face to face interview including 23 questions selected by a previously published questionnaire [23]. The measures investigated at Caretaker level are reported in Table 1 (Caretaker column). The assessments at the Herd level (*n* = 528 camels; Herd column of Table 1) consisted of environmental parameters collected using a weather station (Testo 410-2; Testo Spa, Milan, Italy) as well as the recording of data relating to the characteristics of pens (i.e., size and shape) and facilities, such as shelter and feeding/watering points. In addition to quantitative measures (i.e., number and size), the evaluation of pen facilities included several qualitative measures, such as their cleanliness (classified as “dirty”, “partially dirty”, or “clean”), building material and the placement of the feed and water troughs (in the shade or under the sun), type of feed, and presence of salt blocks. Moreover, the water temperature was taken using a thermometer (Mabis thermometer, Briggs Health Care, West Des Moines, IA, USA) and the presence and the volume of rubbish inside the pens were recorded. Finally, the number of camels showing specific behaviours (e.g., social and abnormal behaviours), manifesting pain induced by procedures (i.e., cauterization, nose-ring, injuries from halters or tethering), presenting a disease (e.g., skin and gastroenteric disorders) or physical injury was noted down and expressed as “proportions of camels per pen”. The assessment at the Animal level (Animal column of Table 1) included a deep visual inspection of randomly selected camels in each pen. Although the protocol of Padalino and Menchetti [22] provided a rigorous determination of the number of animals to be sampled at this level, a maximum of two camels per pen were randomly selected and assessed during our study. Consequently, out of the total population of 528 camels assessed at Herd level, only 132 camels were assessed at Animal level. This was due to constraints in the animal ethics as assessors were only permitted to approach a maximum of two camels per pen for this deeper level of investigation, and in some pens only one camel was kept. During the inspection, specific behaviours (i.e., social interactions, stereotypies, feeding behaviours), disease (i.e., locomotory, skin, and respiratory disorders), pain, and the physical injuries of camels were recorded and expressed as absence or presence. Furthermore, the BCS [24] was estimated and the presence of any restraining systems (i.e., hobbles, tethering) was verified and noted down. The Animal level also included behavioural tests such as approaching and bucket tests [22]. During the approaching test, the camel’s responses to the assessor’s approach were evaluated and then classified as “Positive”, “Neutral” or “Negative”. To perform the bucket test, instead, a bucket with fresh clean water was offered to the camel. The “latency time” (i.e., the time the camel took to approach the bucket after it was placed) and the volume of water drunk by the camel were recorded and then used to calculate the Thirst Index [22]. The protocol measures were collected from both inside and outside the pen in approximately 60 min (Figure 1 and Figure 2).

### 2.3. Data Entry and Processing

The measures collected inside and outside the pens were written down using the recording sheets presented by Padalino and Menchetti [22].

The data were then transferred to an Excel^®^ sheet and categorized. The categorization concerned all levels of assessment and, in particular:

- Answers to open-ended questions of the interview (Caretaker level);
- Building materials of facilities, type of feed, bedding and rubbish, type of disease and physical injury (Herd level);
- Material of hobbles, type of disease and physical injury (Animal level).

Finally, the following indicators derived from the collected measures were calculated: (i) actual space allowance, obtained by dividing the space available by the total number of camels in each pen; (ii) trough space and shaded space allowance, obtained by the ratio between the dimension of the facilities and the number of camels in each pen; (iii) proportions of camels showing a specific behaviour or disease in each pen; and (iv) Thirst Index, by scoring and combining the measures (latency time and the volume of water drunk) collected during the bucket test [22].

### 2.4. Selection of Measures

The protocol by Padalino and Menchetti [22] suggested the collection of 105 measures. However, some measures were excluded as they were not applicable during this field study. Among these, the interview question “How do you rank your understanding of animal welfare?” was excluded as most of the caretakers were unable to answer. Other measures (e.g., criteria that caretakers use to identify a camel in pain or distress, type of food, volume of rubbish, environmental parameters, demographic data) were collected, but it was not possible to assign them a score due to inability to group them on a three-point scale. Finally, some measures (e.g., changes in management according to the season, length of rope used for tethering) were excluded due to incomplete data. In the end, 71 measures were scored (i.e., 13 at Caretaker level, 37 at Herd level, and 21 at Animal level).

### 2.5. Scoring and Aggregation of Measures

**Figure 3:**
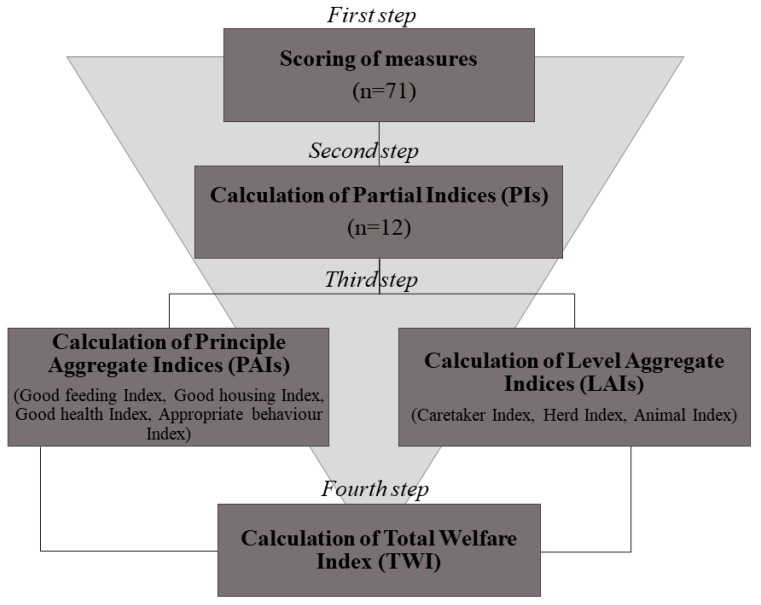
A 4-step process of scoring and aggregation applied to a camel welfare assessment protocol. In the first step, 71 measures collected during the welfare assessment were scored using a 0–2 scale (where 2 was the worst condition). In the second step, the scores were aggregated according to principle and assessment levels and converted on a scale of 0 (worst) to 100 (best) to obtain 12 Partial indices (PIs). In the third step, the PIs were combined into weighted sums to obtain 4 indices aggregated at principle level (PAIs) and 3 indices aggregated at assessment level (LAIs). In the fourth step, the Total Welfare Index (TWI) was obtained by the linear combination of 4 PAIs or 3 LAIs and expressed on the same 0–100 scale.

In order to derive a score for each welfare principle and level of assessment, as well as a total score for each pen, the measures collected during the protocol application were scored and aggregated following a 4-step aggregation process [17,25] (Figure 3).

In the first step, the outcomes of the 71 measures were scored using a 3-point scale: 0 for good welfare, 1 for compromised welfare, and 2 for unacceptable welfare [13,26]. When a welfare measure was instead expressed as a binary response (e.g., presence/absence), only the scores 0 (best situation) and 2 (worst situation) were used [13,26]. Table 2 shows the applied scoring system.

In the second step, the scores of the measures were aggregated according to assessment level and welfare principle and converted into partial indices (PIs). A total of 12 PIs were obtained; namely, in each pen, a score was obtained for Good Feeding at Caretaker, Herd, and Animal level, for Good Housing at Caretaker, Herd and Animal level, and so on. Thus, the measures included in each cell of Table 1 were aggregated into the relative PI. Each PI may vary on a scale from 0 (the worst welfare situation) to 100 (the best welfare situation). Each PI was calculated for each assessment level *i* and each principle *j* as follows:

$${\mathrm{PI}}_{i,j}=100-\left(\frac{\sum_{m=1}^{n_{i,j}}{\left(\mathrm{score}\;\mathrm{of}\;\mathrm{measure}\right)}_{m}\times 100}{k_{i,j}}\right)$$

where:

*i* = assessment level *i*

*j* = principle level *j*

n = number of the measures included in the *j* principle of the *i* level

k = highest possible total score of each principle j within each assessment level *i*.

**Figure 4:**
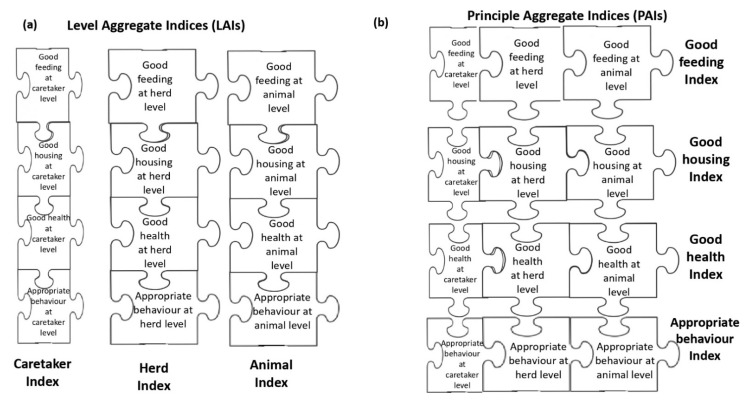
The aggregation of the Partial Indices (PIs) into aggregate indices: (**a**) the puzzle pieces are the PIs aggregated to give the Level Aggregate Indices (LAIs), namely the Caretaker Index, Herd Index, and Animal Index; (**b**) the puzzle pieces are the PIs aggregated to give the Principle Aggregate Indices (PAIs), namely the Good Feeding Index, Good Housing Index, Good Health Index, and Appropriate Behaviour Index. The puzzle pieces representing the measures collected at Caretaker level were smaller than those for the Herd and Animal level because they had less weight (20%) in the calculation of the indices.

In the third step, the PIs were combined to obtain three indices aggregated at assessment level (Level Aggregate Indices, LAIs) regardless of welfare principles. Each pen was therefore scored for Caretaker (i.e., Caretaker Index), Herd (i.e., Herd Index), and Animal level (i.e., Animal Index) on a 0–100 scale. The Animal Index was obtained by averaging the scores of the camels evaluated per pen. Figure 4a shows how the PIs (the puzzle pieces) were aggregated into LAIs. The LAIs expressed the overall assessment obtained by a pen at each assessment level including the four welfare principles with equal weight. The LAI for each assessment level *i* can be calculated as follows:

$${\mathrm{LAI}}_{i}=\left({\mathrm{PI}}_{i,\;Good\;feeding}\times 0.25\right)+\left({\mathrm{PI}}_{i,\;Good\;housing}\times 0.25\right)\;+\left({\mathrm{PI}}_{i,Good\;health}\times 0.25\right)+\left({\mathrm{PI}}_{i,\;Appropriate\;behaviour}\times 0.25\right)$$

where:

*i* = assessment level *i*.

The PIs could also be combined into weighted sums to obtain four indices aggregated at the welfare principle level (Principle Aggregate Indices, PAIs). Thus, each pen was scored for Good Feeding (i.e., Good Feeding Index), Good Housing (i.e., Good Housing Index), Good Health (i.e., Good Health Index), and Appropriate Behaviour (i.e., Appropriate Behaviour Index), regardless of the assessment level. Figure 4b shows how the PIs (the puzzle pieces) were aggregated into PAIs. They could always range from 0 (worst) to 100 (best) [13,17,25]. The PAIs expressed the overall assessment obtained by a pen for each welfare principle including the scores obtained at the three levels of investigations with differential weights. In particular, a lower weight (20%) was attributed to the PIs of Caretaker level as they were based on information reported by the caretaker and not directly collected by the assessor (“questionnaire bias”). The PAI for each principle *j* can be calculated as follows:

$${\mathrm{PAI}}_{j}=\left({\mathrm{PI}}_{Caretaker,\;j}\times 0.20\right)+\left({\mathrm{PI}}_{Herd,\;j}\times 0.40\right)+\left({\mathrm{PI}}_{Animal,\;j}\times 0.40\right)$$

where:

*j* = principle level *j*.

**Figure 5:**
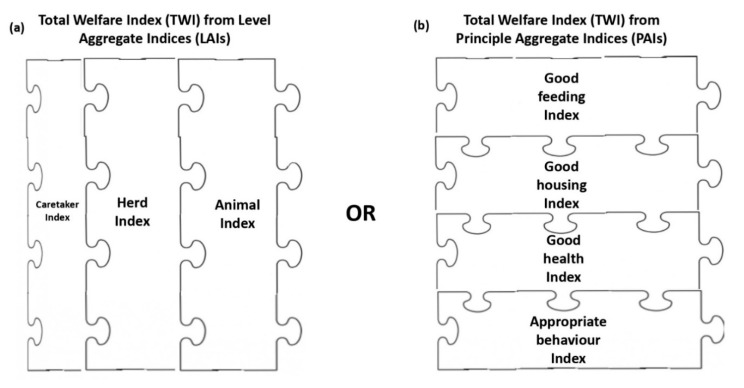
The combination of the aggregate indices into a Total Welfare Index (TWI): (**a**) the puzzle pieces are the Level Aggregate Indices (LAIs), namely the Caretaker Index, Herd Index, and Animal Index; the puzzle pieces of the Caretaker Index were smaller than those of the Herd and Animal Indices because they had less weight in the calculation of the TWI; (**b**) the puzzle pieces are the Principle Aggregate Indices (PAIs), namely the Good Feeding Index, Good Housing Index, Good Health Index, and Appropriate Behaviour Index. The same value of the TWI will be obtained, no matter if we combine LAIs or PAIs.

In the fourth step, the aggregate indices were combined into a weighted sum to obtain the Total Welfare Index (TWI). Each pen had a single TWI which includes all the measures listed in Table 1. The TWI expressed the overall assessment obtained by a pen regardless of the assessment level and welfare principle. The TWI can therefore be obtained, in an equivalent way, both from the combination of the 3 LAIs (Figure 4a) or of the 4 PAIs (Figure 4b). Figure 5 represents the equivalent combination of LAIs (Figure 5a) or PAIs (Figure 5b) to give the TWI.

For the calculation of TWI using the 3 LAIs, differential weights must be attributed as follows:

TWI=(Caretaker Index ×0.20)+(Herd Index×0.40)+(Animal Index×0.40)

The TWI of a pen could also range from 0 (worst welfare condition) to 100 (best welfare condition).

For the calculation of TWI using the 4 PAIs, all PAIs were combined using the same weight (i.e., 25%) as follows:

TWI=(Good feeding Index×0.25)+(Good housing Index × 0.25)+(Good health Index×0.25)+(Appropriate behaviour Index×0.25)

### 2.6. Pen Classification

Two classification systems were proposed based on the PAIs and the TWI, respectively. The PAIs were used to classify the pen, applying a mixed rule system already proposed for the overall assessment of dairy farms [17,27]. This system compares the PAI scores of the pen with predefined reference profiles. The reference profiles for the best and the unacceptable welfare levels of the pens were set at 80 and 20, respectively, in agreement with the Welfare Quality^®^ Network [27]. The limit for the intermediate profile was instead set using the overall mean of the PAIs calculated in our dataset (i.e., 60). Those limits are minimum thresholds that a pen must reach to be included in a category; when the pen does not reach those thresholds, it falls in the lower category. Thus, four welfare classes were identified (Table 3) and the following thresholds were adopted: “excellent”, if the pen scored > 60 for each PAI and >80 for at least 2 of them; “satisfactory”, if the pen scored > 30 for each PAI and >60 for at least 3 of them; “unsatisfactory”, if the pen scored >20 for each PAI and >30 for at least 3 of them; “unacceptable”, if either criteria of unsatisfactory level are not met.

The TWI was instead used to classify the pen by applying statistical binning. In particular, according to TWI tertiles, 3 classes were created and named using a “traffic-light” system: “green light”, if the TWI of the pen was included in the third tertile, “orange light”, if the TWI was included in the second tertile, and “red light” if the TWI was included in the first tertile (Table 3).

### 2.7. Statistical Analysis

Descriptive statistics were used to present the measures and the indices as means and standard deviation (SD), minimum (Min) and maximum (Max), median (Mdn), interquartile range (IQR) or absolute and relative frequencies.

Differences between medians of partial or aggregated indices were analysed using Friedman tests including the pen as a repeated factor, while Dunn’s tests were used to carry out multiple comparisons. These repeated measures analyses were intended to test whether a pen could achieve different scores for the indices of the different assessment and principles levels.

The associations between welfare classes of pens and the scores of measures were evaluated by chi-square or Fisher’s exact tests, while z-tests were used to compare column proportions. The differences in the proportion of animals with physical injuries or diseases between welfare classes were instead evaluated by Mann–Whitney tests. These comparisons concerned the two extreme welfare classes of pens (Satisfactory vs. Unacceptable and Red Light vs. Green Light classes) and aimed to demonstrate the relative importance of some measures on the pen system classifications [17].

Statistical analyses and visualization were performed using SPSS 25.0 (SPSS, an IBM Company, Chicago, IL, USA) and GraphPad Prism, version 7.0 (GraphPad Software, San Diego, CA, USA), respectively. Statistical significance occurred when *p* < 0.05.

## 3. Results

### 3.1. Scoring and Aggregation of Measures

#### 3.1.1. First Step: Scoring of Measures

**Figure 6:**
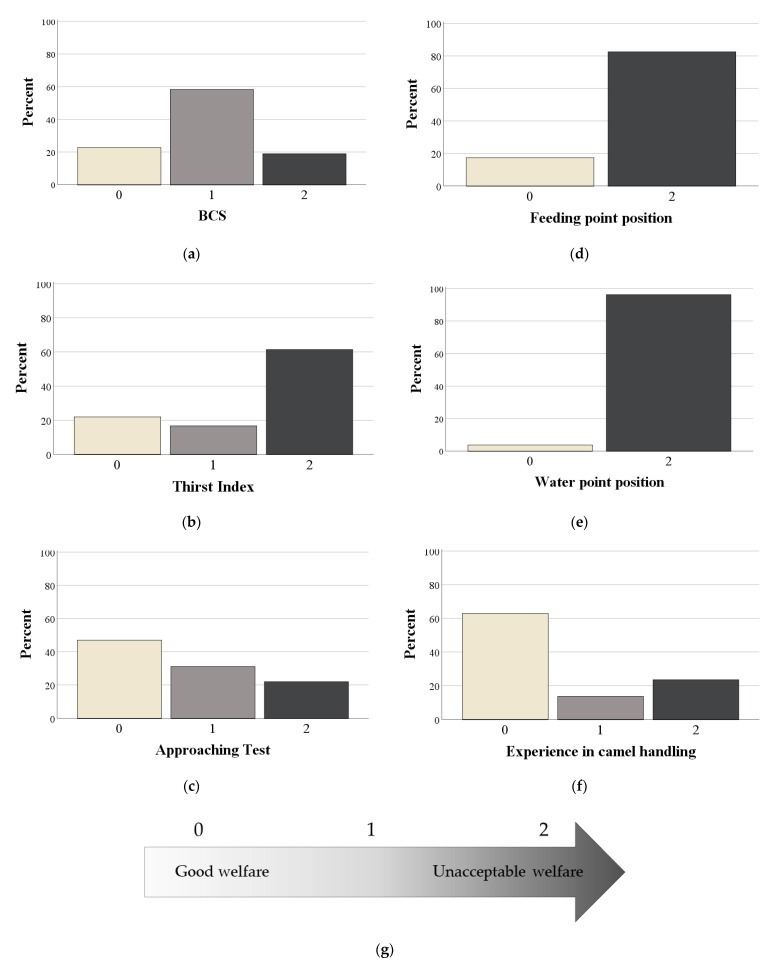
Relative frequencies of selected scores according to some animal and management-based measures, and scoring system: (**a**) BCS; (**b**) Thirst Index; (**c**) approaching test; (**d**) feeding point position; (**e**) watering point position; (**f**) experience in camel handling; (**g**) 3-point scale of the score (0 = good welfare; 1 = intermediate welfare; 2 = unacceptable welfare).

The assignment of the score for the 71 selected measures was intuitive and required some mathematical steps only for continuous variables related to facilities (categorized in tertiles) and for the percentage of animals showing a behaviour or a disease (normalized to the 0–2 range). The results of the descriptive statistics are shown in Tables S1–S6. The scored measures could be graphed to highlight some critical points of the pens. For instance, as shown in Figure 6, most of the assessed camels showed an intermediate score for BCS (i.e., 1), the worst score for the Thirst Index (i.e., 2), and the best score for the Approaching test (i.e., 0). Watering and feeding points were mainly positioned under the sun (score 2), while most of the caretakers had more than 10 years of experience in camel handling (score 0).

#### 3.1.2. Second Step: Calculation of Partial Indices (PIs)

All PIs of Caretaker level, as well as the PI of Good Feeding assessed at Animal level, exhibited high variability (Figure S1). The PIs of Good Feeding obtained the worst median score in all assessment levels (Caretaker level: Mdn = 0.0, IQR = 0.0–50.0; Herd level: Mdn = 26.8, IQR = 17.7–33.8; Animal level: Mdn = 37.5, IQR = 25.0–50.0), followed by Good Health for the Caretaker level, Good Housing for the Herd level, and Appropriate Behaviour for the Animal level (Figure S1). The Friedman test indicated that the PI scores of the pens differed between principles at all assessment levels (*p* < 0.001).

#### 3.1.3. Third Step: Calculation of Aggregate Indices (LAIs and PAIs)

**Figure 7:**
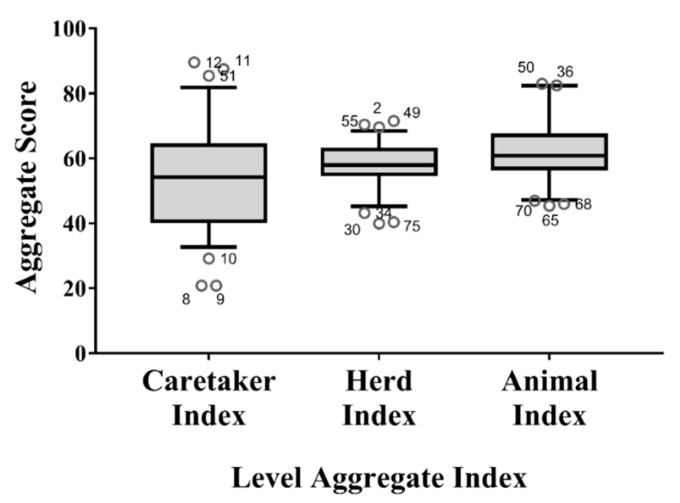
Box plots of the indices aggregated at assessment level (i.e., Caretaker Index, Herd Index and Animal Index). Whiskers define the 5th and 95th percentile while the dots indicate the outliers (scores below the 5th percentile or above the 95th percentile). The identification number of the pen was also reported for each outlier.

The median score of the Caretaker Index was lower than those of the Herd Index (*p* = 0.022) and Animal Index (*p* = 0.002), and showed a greater variability (Figure 7). As shown in Figure 7, there were some outliers (i.e., the pens with very low (below the 5th percentile) or very high scores (over the 95th percentile)).

**Figure 8:**
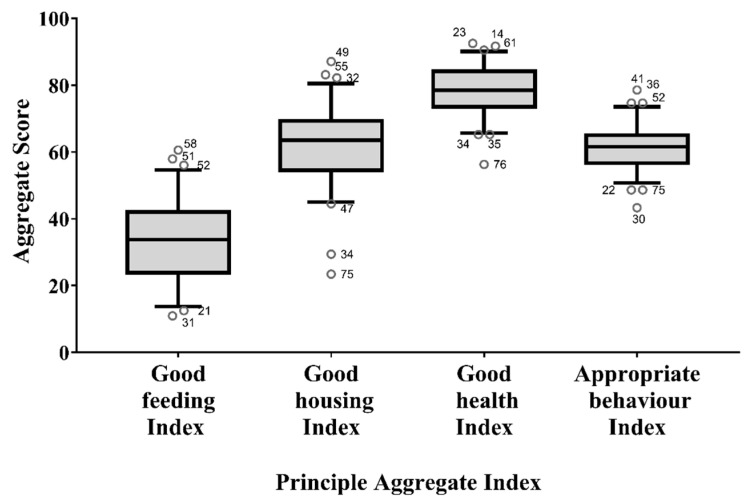
Box plots of the indices aggregated at principle level (i.e., Good Feeding Index, Good Housing Index, Good Health Index, and Appropriate Behaviour Index). Whiskers define the 5th and 95th percentile while the dots indicate the outliers (with scores below the 5th percentile or above the 95th percentile). The identification number of the pen was also reported for each outlier.

Regarding the PAIs, the lowest median score was found for the Good Feeding Index (*p* < 0.001), while the highest was found for the Good Health Index (*p* < 0.001). Outliers were present for all principles (Figure 8).

#### 3.1.4. Fourth Step: Calculation of Total Welfare Index (TWI)

**Figure 9:**
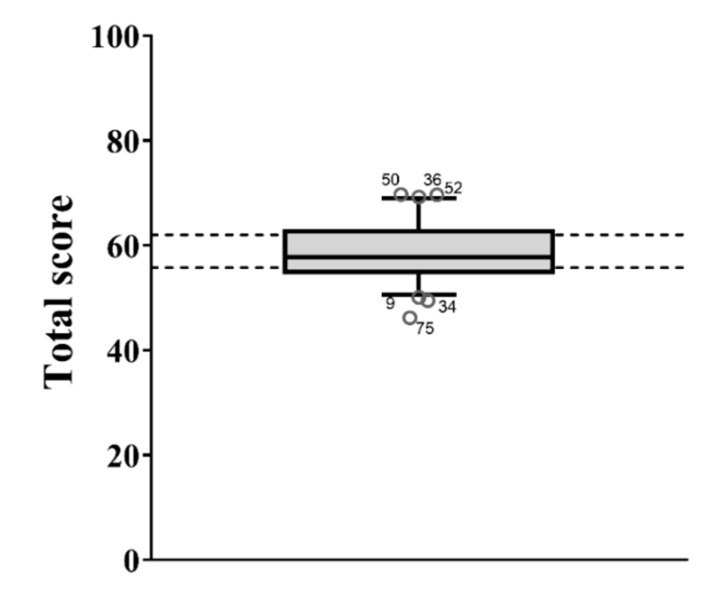
Box plot of the Total Welfare Index. Whiskers of the box define the 5th and 95th percentile while the dots indicate the outliers (below the 5th percentile or over the 95th percentile). The identification number of the pen was also reported for each outlier.

**Figure 10:**
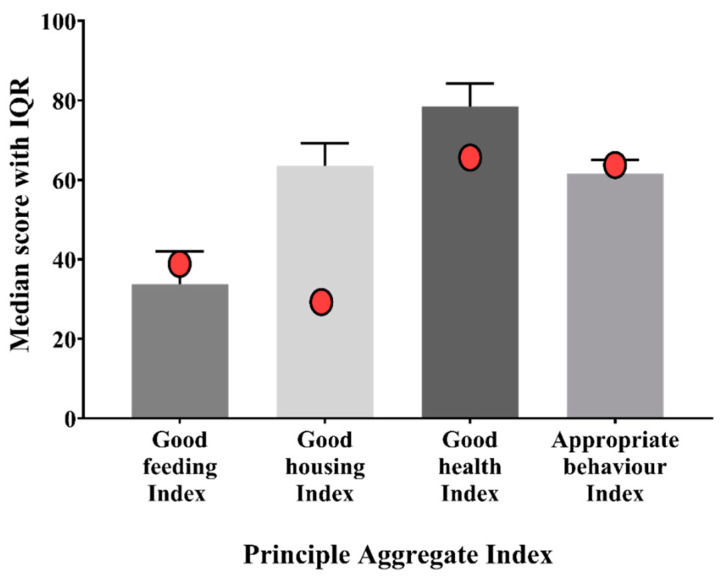
Scores aggregated at the principle level of the pen ID 34 (red dot) in comparison with the median and interquartile range (IQR) of the reference population (all pens of the camel market in Doha). This pen obtained scores below the median of the reference population for the indexes aggregated at Good Housing and Good Health levels.

The TWI ranged from 46.2 to 69.8, with an average score of 58.9 ± 5.3. The box plot (Figure 9) highlighted six pens as outliers. In order to identify the critical aspects of these pens, their PAI scores could be compared with the median values of the reference population. For instance, Figure 10 shows the PAI scores (red dots) of one of the outliers for TWI (pen number ID 34); their positioning in comparison with the median of the reference population identified the principles of Good Housing and Good Health as critical issues.

### 3.2. Pen Classification

#### 3.2.1. Systems of Pen Classification

**Figure 11:**
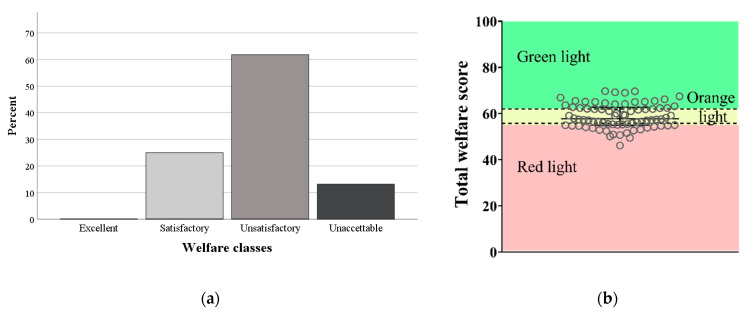
Distribution of pens according to the two classification systems based on scores of the Principle Aggregate Index (relative frequency, Panel (**a**)) and the Total Welfare Index (scatter plot with median and interquartile range, Panel (**b**)), respectively.

In the first classification, which applied rules established *a priori*, most pens fell into the class called “Unsatisfactory”. In particular, the pens were distributed as follows: Excellent (0/76), Satisfactory (19/76), Unsatisfactory (47/76), and Unacceptable (10/76; Figure 11a). In the second classification, the identified thresholds for the TWI were 56.0 and 62.0. Then, the pens were distributed as follows: Red Light (25/76, 32.9%; TWI ≤ 56.0), Orange Light (28/76, 36.8%; 56.0 < TWI ≤ 62.0), and Green Light (23/76, 30.3%; TWI > 62.0; Figure 11b).

The two classification systems partially disagreed for some pens. For example, the pen ID 34 and the pen ID 3 were both classified as Unsatisfactory using the profile of their PAIs (Figure S2; panels on the left), but they were classified as Red Light and Green Light, respectively, according to their TWI scores (panels on the right; TWI = 49.5 and TWI = 65.6 for pens ID 34 and 3, respectively). The pen ID 33 was classified as Unacceptable according to the PAIs, as not all PAIs were >20, but as Orange Light according to its TWI (TWI = 59.3; Figure S2).

#### 3.2.2. Relative Importance of the Measures on the Pen System Classifications

Table 4 and Table 5 show the results of the comparison of some animal- and management-based measures between pens included in the two extreme classes. Pens classified as Unacceptable or Red Light showed fewer camels in optimal physical condition (i.e., score 0 for BCS) than pens classified as Satisfactory (*p* = 0.002) or Green Light (*p* = 0.014). Both classification systems also showed differences in the distribution of the Thirst Index: pens classified in the worst classes had more camels showing the worst score (i.e., score 2, *p* < 0.05). However, only the z-test was significant for the classification according to the profiles of PAIs (Fisher-exact *p* < 0.1; z-test *p* < 0.05; Table 4). Differences in the intermediate score of the approaching test (z-test *p* < 0.05) and water space per animal (Fisher-exact and z-test *p*-values < 0.05) were also found. Moreover, a higher proportion of pens without shelter (score 2) were classified as Unacceptable (z-test *p*-value < 0.05). A highly significant association was found between pens classified as Unacceptable and the lowest frequency of water distribution (score 2, *p* = 0.005). In addition to BCS and Thirst Index, a higher proportion of animals with a disease (*p* = 0.028) or with physical injuries (*p* = 0.007) were present in pens classified as Red Light (Table 5). Moreover, in these pens, the feeding was not offered ad libitum (*p* = 0.046) and the bedding was scored as dirty (*p* = 0.027).

## 4. Discussion

This is the first study applying a protocol for assessing the welfare status of camels kept under intensive conditions. The welfare measures included in the protocol proposed by Padalino and Menchetti [22] were firstly collected at the camel market in Doha; data were then selected, interpreted, and processed into aggregated welfare indices. Finally, these indices were used to classify camel units (i.e., pens) according to their welfare levels. The proposed model allowed us to identify factors of concern and possible hazards for camel welfare. Even though this model showed some limitations and should be further tested and improved, it relied on intuitive and feasible approaches. Our model needs to be implemented by camel industry members using the results of camel welfare assessments in different farms and markets worldwide. Our study is the first step in the process of developing camel welfare standards.

The proposed model included a 4-step aggregation process, resulting in an overall welfare index (i.e., TWI). The first step of this process consisted of scoring the measures collected from each pen at the market. The attribution of the score on a 0–2 scale was intuitive and in accordance with the systems proposed for other animal species [13,26,28]. A simple graphical representation of the scored measures was useful for the identification of some camel welfare concerns and hazards [29]. Among the animal-based measures, the Thirst Index received the worst score in most of the pens, suggesting that “prolonged thirst” was the most common poor welfare outcome at the Doha market. The lack of watering points or their placement in the sun could be therefore identified as a hazard for the prolonged thirst of camels. In agreement with our findings, Bergin and Nijman [30] found that animals kept in Moroccan markets showed poor welfare conditions due to lack of water access, sun/heat protection, and facilities to hide from stressors. Similar welfare issues have been found with cattle kept at markets in South America [31] and small ruminants in a large abattoir in Ethiopia [32]. Pritchard et al. [33], using skin tenting in a large sample of equids in the Middle East, found that 37% of donkeys and 50% of horses showed signs of dehydration. Water accessibility and quality are features of paramount importance for animal welfare and, in particular, in regions characterized by hot and arid climate, animal handlers must pay attention to watering management.

Another area of concern was related to feeding management. The scored measures in the present protocol showed, indeed, that the body conditions of the camels were not always optimal, as almost 60% received an intermediate score. However, it is worth highlighting that more alarming results were found in equids reared in the same geographical area (70% were underweight) [33] and in South Asia (80% were underweight) [34]. In our scoring system, the worst score for BCS was attributed to both underweight and obese camels. Obesity is, indeed, a growing concern for camel welfare, as reported for dairy camels in the United Arab Emirates (Dr. Abdul Raziq, unpublished). Obesity predisposes camels to diabetes mellitus [35] and, as in other species [36,37], could lead to metabolic, locomotory, and reproductive disorders, so it should always be considered as a welfare concern. However, it is worth noting that the proposed 3 point scale for BCS is not suitable for risk analysis as the worst score included two opposite welfare concerns (cachexia and obesity). Camel handlers should be educated on appropriate feeding management.

The second step of the proposed model consisted of calculations to obtain Partial Indices (PIs). Each PI was expressed on a scale of 0 (the worst situation) to 100 (the best situation), in agreement with the indices proposed in the literature for other animal species [14,17,19,25,38]. These PIs may be an effective tool to identify areas of concern and hazards, allowing the farmers to apply corrective actions for the improvement of their camels’ welfare. Thus, caretakers and owners could focus their efforts on only improving the aspects of their management that obtained low PI scores. In our study, the principle of Good Feeding obtained the worst median score in all assessment levels. This finding suggests that an insufficient score in terms of welfare was obtained for all measures included in the principle of Good Feeding, regardless of the assessment level (namely Thirst Index and BCS evaluated at the Animal level, the facilities evaluated at Herd level, and the watering and feeding management evaluated at Caretaker level). Corrective actions in the Doha market should therefore be implemented in operations such as trough number and position, water and feed quality, and the frequency of water and feed distribution.

In the third step, the PIs were combined to obtain three indices aggregated at assessment level (LAIs) and four indices aggregated at principle level (PAIs). The analysis of the LAIs showed that the pens achieved the lowest scores for the Caretaker Index. A review of the measures included in this assessment level could be advisable, but this result could also confirm the lack of involvement of caretakers in welfare-related issues. The analysis of PAIs showed, instead, that pens obtained the best score for the Good Health Index. Health conditions, therefore, did not appear to be a major concern for the camels kept at the Doha market. The free veterinary service offered in Qatar could be one of the contributing factors explaining the good health conditions of the considered camels. However, it can also be assumed that only healthy camels were brought to the market to be sold, or sick camels were sent to slaughter and no longer kept at the market. Within the principle of Good Health, the worst scores were obtained for measures indicating skin disorders, physical injuries, cauterizations, and scars from hobbles. These findings are in agreement with epidemiological studies reporting dermatitis, in particular mange, and skin wounds as the most common clinical conditions among camels in Saudi Arabia and Sudan [39,40]. Similarly, the presence of many cauterizations was not surprising as they are commonly used to treat many camels’ pathologies [41]. A higher prevalence of skin wounds, mainly due to incorrect applications of restraint tools, was also found in working equids in Egypt [42], India [43], and Nepal [34]. Training of the caretakers, better housing conditions and better management practices, such as the use of pads under the hobbles, could reduce the risk of injury in camels and improve their welfare level. Contrary to what was found for the Good Health Index, the Good Feeding Index was extremely low in most pens. This finding confirms those already discussed for PIs and individual measures, highlighting that feeding and watering management require corrective actions. Our results were in agreement with the literature. Good Feeding also had the lowest score in dairy farms in Algeria [44], and Gebremedhin et al. [45] identified inappropriate shelter, feed and water supply as issues at livestock markets in Ethiopia. However, as shown by other studies [25,38,46,47], farm animal welfare problems can be highly variable, since they depend on many factors such as species, environment and farming systems, so our data are valid only for the examined camel market.

In the final step, the aggregated indices were combined to obtain the Total Welfare Index (TWI). The TWI offers an overall welfare assessment of the pens, enabling the identification of the pens with the worst and the best welfare levels (the lowest and highest TWI scores). The TWI score could be used to implement systems of voluntary certification or to reward greater welfare performance within a population [3]. Conversely, the pens with the lowest TWI scores are those deserving further attention. For those pens, a backward analysis of the scored measures, aggregate and partial indices is required to identify camel welfare concerns and hazards.

Two models were proposed to classify camel pens. The first ranked the pens based on TWI scores and used a “traffic-light” system to label welfare classes. The other classification system used, instead, the profiles of PAIs as proposed for dairy farms [8,17,27]. According to PAI profiles, most of the pens at the camel market were classified as “Unsatisfactory”, a few pens were “Unacceptable”, and no pens were “Excellent”. This was expected and in agreement with what was found in dairy farms [17,44]. The two classification systems did not always converge because, for example, a pen may achieve a good TWI score even if one or more PAIs are below the predefined threshold level. As a result, some pens were classified as Unsatisfactory by one system but received Green Light by the other. As suggested above, the use of the TWI does not allow the direct identification of critical points and does not take into consideration the multidimensional concept of welfare [27]. Furthermore, the bins identified for the TWI could only be used to rank our reference population and require external validation. The profiles of the PAIs are instead established a priori. Thus, the model based on PAIs seems to be more efficient, producing an absolute score for any animal unit [18] and being very flexible. Moreover, the welfare classes obtained using the PAI profiles reflected the multi-dimensional nature of welfare and the relative importance of various welfare principles [17,18,27]. This classification system, finally, could lead to the improvement of specific deficient measures within the pen, directing attention only on the measures that require improvement [17]. The model based on the PAI profile, therefore, seems the most appropriate for classifying camel pens, both from a conceptual and a practical point of view.

The relative importance of the measures on the pen classification produced similar results for both systems. The animal-based measures with a strong influence on classification were BCS and Thirst Index. The incidence of disease and physical injuries was also higher in the worst pens when compared with the pens included in the best classes. The most important management-based measures were, instead, the frequency of feeding and water distribution, the water space, the presence of a shelter, and the cleanliness of bedding. These findings, therefore, identified the most important hazards (i.e., factors with the potential to cause poor welfare [29]) for pens with low levels of well-being. They confirmed the report of Gebremedhin et al. [45] but also emphasized the role of the cleanliness of the bedding for safeguarding camel welfare. Further studies concerning an analysis of the risks could help in the selection of measures to refine the protocol, making it easier to put into practice.

### Limitations and Further Technical Considerations on the Selection of Measures, Scoring System and Aggregation Process

Some measures proposed by Padalino and Menchetti [22] were not included in the overall welfare indices for several reasons. The question “How do you rank your understanding of animal welfare?”, for example, was not included because many caretakers had difficulty understanding the concept of animal welfare. Linguistic, social, and cultural factors could, indeed, influence the interpretation of this term [48]. Different people may also interpret animal rights and animal welfare differently as a result of peculiar human-animal-environment relationships. This may be our case; it is indeed worth highlighting that, at the Doha market, caretakers often shared living spaces with camels. The lack of plausible answers to this question also suggests that camel caretakers need to be educated on welfare aspects, and a more animal welfare friendly approach to camel farming needs to be further promoted. Other measures, instead, could not be included in the overall welfare indices as the lack of standard references did not allow their scoring. Some of these measures could be removed from the recording sheets to simplify the assessment protocol (e.g., the trough material and type of fence). Other measures, such as environmental parameters, the demographic data of caretakers or the volume of rubbish, need instead further analysis to evaluate their influence on camel welfare. Unlike other livestock species [49,50], for example, there are no studies evaluating the effect of Temperature Humidity Index on the level of heat stress of the camel.

The measures included in the overall welfare indices were scored using different approaches, some of which deserve further discussion. For the scoring of some continuous measures, such as space allowance and feeding space, statistical binning was chosen. This is an objective method used to categorize continuous variables when there is no bibliography available [51,52], but the thresholds calculated in the present study require external validation in different contexts. The score for the proportion of camels showing disease or specific behaviours was instead obtained by their conversion on the 0–2 range. It was easy to calculate and intuitive, but it differed from the method used in other assessment protocols. Welfare Quality^®^ protocols [11,13] proposed, instead, specific indices for these measures, weighing the proportion of animals for the severity of the manifestations and applying predefined thresholds and I-spline functions. The method proposed by Welfare Quality^®^ is refined and efficient, but it could be difficult to use for non-experts as well as difficult to transpose to other animal species. Thus, the approach of the present study mainly favoured feasibility and objectivity to help a large-scale replication.

The last considerations concern the aggregation process. First, unlike the model applied for the Welfare Quality^®^ protocol [17,19], the present approach did not provide a preliminary aggregation of measures into the 12 welfare criteria. It indeed maintained the setting of Padalino and Menchetti’s protocol [22], which stratified the measures for assessment levels and welfare principles. Second, the mathematical calculation of PIs was simple as the measures were not weighted. This could also be a limit of the present study because all measures had the same impact [18]. On the other hand, the assignment of a relative weight to an individual measure is a subjective technique [2] and requires a long process of data analysis, the involvement of many stockworkers as well as good mathematical skills [2,47]. The weighing of scores therefore seems premature for camel species and would affect the feasibility, repeatability, and lay use of the protocol [2]. Third, the different number of measures included in the PIs may result in double-counting or inaccuracy. Thus, the measures included in some PIs should be refined and integrated. However, this refining and integration process might be possible only after having applied the model on larger datasets. Only the application of the proposed method on a variety of camel farms and the collection of data by a large number of assessors will permit its validation.

## 5. Conclusions

Notwithstanding the limitations mentioned above, the proposed protocol and model for the analysis of the data were easy to apply. The refined measures and the proposed scoring, aggregated indices and method of pen classification were able to identify welfare concerns and hazards in camels kept in pens at a market in Qatar. Further applications of the welfare protocol and the proposed scoring systems are needed to refine them and to collect a large dataset, which is crucial to define welfare standards in camels.

## Figures and Tables

**Table 1:** Synthesis of the measures collected in each pen during the welfare assessment according to welfare principles and level of investigation, as proposed by the protocol of Padalino and Menchetti [22].

Welfare Principle	Level of Investigation		
	Caretaker	Herd	Animal
**Good Feeding**	Feeding and watering management	Feeding and watering points (number, dimension, location) Feed and water availability Feed and water quality Feeding and watering space per animal Presence of salt Proportion of camels drinking, eating and ruminating	Body Condition Score (BCS) Thirst Index
**Good Housing**	Caretaker’s experience in working with animals Number of animals handled by the caretaker in the busiest week	Space allowance Shelter (presence and shaded space allowance) Fence condition Bedding (presence and cleanliness) Rubbish (presence and dimension) Proportion of camels hobbled and in shade	Resting behaviour Location (under the sun/in shade) Insects (quality, quantity) Tethering Hobbled
**Good Health**	Past camel disease observed Camel health check Medical treatments	Proportion of camels with disease, physical injuries, scars from hobbles, cauterization, nose-ring Proportion of camels in pain	Presence of disease, physical injuries, locomotory disorders, skin disorders, discharge, mastitis or abnormal udder, respiratory disorders, pain
**Appropriate Behaviour**	Caretaker’s experience in camel handling Current behavioural problems Caretaker’s skills in identifying distress	Proportion of camels resting, standing quietly, aggressive Proportion of camels showing stereotypies and other abnormal behaviour	Social interaction Stereotypies Abnormal behaviour Feeding and rumination Approaching test

**Table 2:** Scoring system developed for the measures included in the camel welfare protocol by Padalino and Menchetti [22].

Measure		Criteria	Scores
Who carries out health assessment or medical treatment		A veterinarian	0
		A non-veterinarian	1
		Not conducted	2
Grade of caretaker’s ability in identifying a camel in distress/pain		High–Very high	0
		Moderate	1
		Low–Some	2
Years of caretaker’s experience		>10 years	0
		6–10 years	1
		0–5 years	2
Food/water distribution		Ad libitum	0
		Rationed	2
Food/water position ^1^		In the shade	0
		In the sun	2
Continuous variables related to facilities ^1,2^		Statistical binning (tertiles)	0 (best situation)
			1 (second tertile group)
			2 (worst situation)
Cleanliness of facilities ^1^		Clean	0
		Partially Dirty	1
		Dirty	2
Presence of salt block, shelter, shade, bedding		Yes	0
		No	2
Presence of rubbish, broken fence, insects		No	0
		Yes	2
Body Condition Score (BCS)		3 (good body condition)	0
		2, 4 (moderate body condition)	1
		0–1, 5 (poor body condition, lean or obese)	2
Thirst Index		0	0
		1	1
		2–3	2
Presence of a disease, physical injuries, pain or behaviour indicating poor welfare ^3^			
	Animal level	No	0
		Yes	2
	Herd level	Percentage of animals with the disease/injury/pain/behaviour	0 (0%)–2 (100%)
Presence of behaviour indicating good welfare ^4^			
	Animal level	Yes	0
		No	2
	Herd level	Percentage of animals showing the behaviour	0 (100%)–2 (0%)
Tethering/Hobbled		No	0
		Yes	2
Responses during the approaching test		Positive	0
		Neutral	1
		Negative	2

^1^ when more than one trough was present in the pen, the score was attributed to a randomly chosen one. ^2^ dimension and number of troughs, water temperature, space allowance, trough space, shaded space allowance. ^3^ aggressive behaviours, stereotypies, and other abnormal behaviours. ^4^ resting, standing quietly, positive social behaviours, feeding, rumination.

**Table 3:** Two classification systems proposed to classify the pens where camels were kept. The two systems were based on the profiles of the 4 Principle Aggregate Indexes (PAIs) and the tertiles of Total Welfare Index (TWI), respectively.

Parameter	Criteria	Welfare Classes
Principle Aggregate Indexes	>60 for each principle and >80 for 2 principles	Excellent
	>30 for each principle and >60 for 3 principles	Satisfactory
	>20 for each principle and >30 for 3 principles	Unsatisfactory
	Failure to meet the above requirements	Unacceptable
Total Welfare Index	Third tertile	Green Light
	Second tertile	Orange Light
	First tertile	Red Light

**Table 4:** Number and percentage or median and interquartile range of animal- and management-based measures recorded in the pens classified as Satisfactory (*n* = 19 pens) and Unacceptable (*n* = 10 pens) according to the profiles of Principle Aggregate Indices (PAIs). The measures, except the proportion of animals, were scored on a 0 (good welfare)–2 (unacceptable welfare) scale.

Type of Measure	Measure	Score	Welfare Class				*p* Value *
			Satisfactory		Unacceptable		
			Count	N %	Count	N %	
Animal-based	**BCS**	0	12 ^a^	63.2%	0 ^b^	0.0%	0.002
		1	3 ^a^	15.8%	5 ^a^	50.0%	
		2	4 ^a^	21.0%	5 ^a^	50.0%	
	**Thirst Index**	0	6 ^a^	31.6%	0 ^b^	0.0%	0.088
		1	3 ^a^	15.8%	1 ^b^	10.0%	
		2	10 ^a^	52.6%	9 ^b^	90.0%	
	**Approaching test**	0	15 ^a^	78.9%	5 ^a^	50.0%	0.079
		1	1 ^a^	5.3%	4 ^b^	40.0%	
		2	3 ^a^	15.8%	1 ^a^	10.0%	
	Camels with a disease ^1^		33.3 (0.0–50.0)		36.7 (33.3–60.0)		0.403
	Camels with physical injuries ^1^		0.0 (0.0–0.0)		7.1 (0.0–20.0)		0.138
Resourced- and management-based	Frequency of feed distribution ^2^	0	5 ^a^	26.3%	0 ^a^	0.0%	0.134
		2	14 ^a^	73.7%	10 ^a^	100.0%	
	**Frequency of water distribution ^2^**	0	10 ^a^	52.6%	0 ^a^	0.0%	0.005
		2	9 ^a^	47.4%	10 ^b^	100.0%	
	**Water space per animal**	0	8 ^a^	42.1%	2 ^a^	20.0%	0.048
		1	4 ^a^	21.1%	7 ^b^	70.0%	
		2	7 ^a^	36.8%	1 ^a^	10.0%	
	Feeding space per animal	0	7 ^a^	36.8%	2 ^a^	20.0%	0.560
		1	6 ^a^	31.6%	3 ^a^	30.0%	
		2	6 ^a^	31.6%	5 ^a^	50.0%	
	**Shelter ^3^**	0	19 ^a^	100.0%	8 ^a^	80.0%	0.111
		2	0 ^a^	0.0%	2 ^b^	20.0%	
	Cleanliness of bedding	0	9 ^a^	47.4%	4 ^a^	40.0%	0.213
		1	9 ^a^	47.4%	3 ^a^	30.0%	
		2	1 ^a^	5.2%	3 ^a^	30.0%	
	Rubbish ^3^	0	12 ^a^	63.2%	3 ^a^	30.0%	0.128
		2	7 ^a^	36.8%	7 ^a^	70.0%	

BCS = Body Condition Score. * *p*-value for chi-square, Fisher’s exact or Mann–Whitney tests. Values in the same row followed by a different letter (^a^ or ^b^) differ significantly (*p* < 0.05; z-test). Measures in bold denote significant variables at the *p* < 0.05 level by chi-square, Fisher’s, Mann–Whitney and/or z-tests. ^1^ as proportion of animals, expressed as median and interquartile range. ^2^ rationed versus ad libitum. ^3^ presence versus absence.

**Table 5:** Number and percentage or median and interquartile range of animal- and management-based measures recorded in the pens classified as Green (*n* = 23 pens) and Red Light (*n* = 25 pens) according to the tertiles of the Total Welfare Index. The measures, except the proportion of animals, were scored on a 0 (good welfare)–2 (unacceptable welfare) scale.

Type of Measure	Measure	Score	Welfare Class				*p* Value *
			Green		Red		
			Count	N %	Count	N %	
Animal-based	**BCS**	0	13 ^a^	56.5%	4 ^b^	16.0%	0.014
		1	7 ^a^	30.4%	16 ^b^	64.0%	
		2	3 ^a^	13.1%	5 ^a^	20.0%	
	**Thirst Index**	0	10 ^a^	43.5%	2 ^b^	8.0%	0.011
		1	4 ^a^	17.4%	4 ^a^	16.0%	
		2	9 ^a^	39.1%	19 ^b^	76.0%	
	Approaching test	0	14 ^a^	60.9%	11 ^a^	44.0%	0.519
		1	5 ^a^	21.7%	7 ^a^	28.0%	
		2	4 ^a^	17.4%	7 ^a^	28.0%	
	**Camels with a disease ^1^**		33.3 (0.0–50.0)		66.7 (31.0–92.3)		0.028
	**Camels with physical injuries ^1^**		0.0 (0.0–0.0)		6.3 (0.0–20.0)		0.007
Resource- and management-based	**Frequency of feed distribution ^2^**	0	9 ^a^	39.1%	3 ^b^	12.0%	0.046
		2	14 ^a^	60.9%	22 ^b^	88.0%	
	Frequency of water distribution ^2^	0	13 ^a^	56.5%	11 ^a^	44.0%	0.564
		2	10 ^a^	43.5%	14 ^a^	56.0%	
	Water space per animal	0	1 ^a^	47.8%	9 ^a^	36.0%	0.439
		1	5 ^a^	21.8%	10 ^a^	40.0%	
		2	7 ^a^	30.4%	6 ^a^	24.0%	
	Feeding space per animal	0	9 ^a^	39.1%	7 ^a^	28.0%	0.449
		1	8 ^a^	34.8%	7 ^a^	28.0%	
		2	6 ^a^	26.1%	11 ^a^	44.0%	
	Shelter ^3^	0	21 ^a^	91.3%	20 ^a^	80.0%	0.419
		2	2 ^a^	8.7%	5 ^a^	20.0%	
	**Cleanliness of bedding**	0	9 ^a^	39.1%	10 ^a^	40.0%	0.027
		1	13 _a_	56.5%	7 ^b^	28.0%	
		2	1 ^a^	4.4%	8 ^b^	32.0%	
	Rubbish ^3^	0	14 ^a^	60.9%	11 ^a^	44.0%	0.265
		2	9 ^a^	39.1%	14 ^a^	56.0%	

BCS = Body Condition Score. * *p*-value for chi-square, Fisher’s exact or Mann–Whitney tests. Values in the same row followed by a different letter (^a^ or ^b^) differ significantly (*p* < 0.05; z-test). Measures in bold denote significant variables at the *p* < 0.05 level by chi-square, Fisher’s, Mann–Whitney and/or z-tests. ^1^ as proportion of animals, expressed as median and interquartile range. ^2^ rationed versus ad libitum. ^3^ presence versus absence.

## Data Availability

Data are contained within the article or Supplementary Materials.

## Supplementary Materials

The following are available online at https://www.mdpi.com/2076-2615/11/2/494/s1, Figure S1: Partial indices (PIs) of the 4 welfare principles for Caretaker (a), Herd (b), and Animal level (c). Whiskers define the 5th and 95th percentile while the dots indicate the outliers (scores below the 5th percentile or above the 95th percentile), Figure S2: Classification of three pens (ID 34, ID 3, and ID 33) according to the indices aggregated at principle level and the bins of the Total Welfare Index. The class of each pen is circled in red for each classification system, Table S1: Absolute (N) and relative (N%) frequency distribution of scores of the measures collected at Caretaker level according to the welfare principle (Good Feeding, Good Housing, Good Health, and Appropriate Behaviour). The possible range for the total score of each principle is also reported, Table S2: Absolute (N) and relative (N%) frequency distribution or mean and standard deviation (SD) of scores of the measures collected at Herd level for the principle of Good Feeding. The possible range for the total score is also reported, Table S3: Absolute (N) and relative (N%) frequency distribution or mean and standard deviation (SD) of scores of the measures collected at Herd level for the principle of Good Housing. The possible range for the total score is also reported, Table S4: Mean and standard deviation (SD) of scores of the measures collected at Herd level for the principles of Good Health and Appropriate Behaviour. The possible range for the total score of each principle is also reported, Table S5: Absolute (N) and relative (N%) frequency distribution of scores of the measures collected at Animal level for the principles of Good Feeding and Good Housing. The possible range for the total score of each principle is also reported, Table S6: Absolute (N) and relative (N%) frequency distribution of scores of the measures collected at Animal level for the principles of Good Health and Appropriate Behaviour. The possible range for the total score of each principle is also reported.
