# Supplementary material for: Application of a Protocol to Assess Camel Welfare: Scoring System of Collected Measures, Aggregated Assessment Indices, and Criteria to Classify a Pen
Source: Animals (Basel). 2021 Feb 13;11(2):494. doi: 10.3390/ani11020494 (PMC7918070; doi:10.3390/ani11020494)
Supplement: Supplementary file 1 [file animals-11-00494-s001.pdf]

Table S1. Absolute (N) and relative (N%) frequency distribution of scores of the measures collected at Caretaker level according to the welfare principle (Good feeding, Good housing, Good health, and Appropriate behaviour). The possible range for Total score of each principle is also reported.

Measure	Criteria	Score	N	N %
Frequency of feed distribution	Ad libitum	0	15	19.7%
	Rationed	2	61	80.3%
Frequency of water distribution	Ad libitum	0	35	46.1%
	Rationed	2	41	53.9%
**Total score Good feeding: 0-4**				
Years of experience in working with camels	>10 ys	0	36	47.4%
	6-10	1	22	28.9%
	0-5 ys	2	18	23.7%
Camels reared at the farm in the busiest week	0-10 camels	0	25	32.9%
	11-30 camels	1	41	53.9%
	31-50 camels	2	10	13.2%
**Total score Good housing: 0-4**				
Who assesses the health of the camels	Vet	0	39	51.3%
	No vet	1	31	40.8%
	Not conducted	2	6	7.9%
Who treats the camel when it is sick	Vet	0	63	82.9%
	No vet	1	13	17.1%
Who administers vaccinations to camels	Vet	0	10	13.2%
	No vet	1	15	19.7%
	Not conducted	2	51	67.1%
Who administers endoparasite treatments to camels?	Vet	0	34	44.7%
	No vet	1	40	52.6%
	Not conducted	2	2	2.6%
Who administers ectoparasite treatments to camels	Vet	0	15	19.7%
	No vet	1	54	71.1%
	Not conducted	2	7	9.2%
Health problems have been observed in camels over the last year	No	0	9	11.8%
	Yes	2	67	88.2%
**Total score Good health: 0-12**				
Years of experience in camel handling	>10 ys	0	46	60.5%
	6-10	1	11	14.5%
	0-5 ys	2	19	25.0%
Camels show behavioural problems	No	0	35	46.1%
	Yes	2	41	53.9%
Caretaker's ability in identifying a camel in distress/pain	High- very high	0	57	75.0%
	Moderate	1	19	25.0%
	Some - Low	2	0	0.0%
**Total score Appropriate behaviour: 0-6**				

Table S2. Absolute (N) and relative (N%) frequency distribution or mean and standard deviation (SD) of scores of the measures collected at Herd level for the principle of Good feeding. The possible range for Total score is also reported.

Measure	Criteria	Score	N	N %
Number of water points	≥2 water points	0	5	6.6%
	1 water points	1	64	84.2%
	0 water points	2	7	9.2%
Water availability	Yes	0	59	77.6%
	No	2	17	22.4%
Water point dimension*	>0.50 m²	0	25	32.9%
	0.46-0.50 m²	1	26	34.2%
	≤0.45 m²#	2	25	32.9%
Water point location*	Shade	0	3	3.9%
	Sun	2	73	96.1%
Water temperature*	≤34.5 °C	0	20	26.3%
	34.6-37.5 °C	1	20	26.3%
	>37.5 °C#	2	36	47.4%
Water quality*	Clean	0	34	44.7%
	Partially dirty	1	17	22.4%
	Dirty #	2	25	32.9%
Water space per animal	>0.160 m²/camel	2	22	28.9%
	0.061-0.160 m²/camel	1	27	35.5%
	≤0.060 m²/camel#	0	27	35.5%
Proportion of drinking camels (mean±SD)	0 (100% animals drinking)-<br>2 (0% animals drinking)		1.96±0.20	
Number of feeding points	>2 feeding points	0	27	35.5%
	1-2 feeding points	1	48	63.2%
	0 feeding points	2	1	1.3%
Feed availability	Yes	0	47	61.8%
	No	2	29	38.2%
Feeding point dimension*	>2.50 m²	0	23	30.3%
	1.51-2.50 m²	1	27	35.5%
	≤1.50 m²#	2	26	34.2%
Feeding point location*	Shade	0	13	17.1%
	Sun	2	63	82.9%
Feed quality*	Clean	0	40	52.6%
	Partially dirty	1	6	7.9%
	Dirty #	2	30	39.5%
Salt (presence)	Yes	0	16	21.1%
	No	2	60	78.9%
Feeding space per animal	>1.10 m²/camel	0	26	34.2%
	0.41-1.10 m²/camel	1	24	31.6%
	≤0.40 m²/camel	2	26	34.2%
Proportion of eating camels (mean±SD)	0 (100% animals eating)-<br>2 (0% animals eating)		1.69±0.51	
Proportion of ruminating camels (mean±SD)	0 (100% animals ruminating)-<br>2 (0% animals ruminating)		1.70±0.43	
**Total score Good feeding: 0-34**				

*referred to a randomly selected trough (when more than one trough was present)
or not available

Table S3. Absolute (N) and relative (N%) frequency distribution or mean and standard deviation (SD) of scores of the measures collected at Herd level for the principle of Good housing. The possible range for Total score is also reported.

Measure	Criteria	Score	N	N %
Space allowance	>40.0 m²/animal	0	25	32.9%
	19.1-40.0 m²/animal	1	25	32.9%
	≤19.0 m²/animal	2	26	34.2%
Shelter (presence)	Yes	0	65	85.5%
	No	2	11	14.5%
Shaded space allowance	>7.00 m²/animal	0	26	34.2%
	2.51-7.00 m²/animal	1	27	35.5%
	≤2.50[#] m²/animal	2	23	30.3%
Broken fences	No	0	13	17.1%
	Yes	2	63	82.9%
Bedding (presence)	Yes	0	76	100.0%
	No	2	0	0.0%
Cleanliness of bedding	Clean	0	30	39.5%
	Partially dirty	1	34	44.7%
	Dirty	2	12	15.8%
Rubbish (presence)	No	0	38	50.0%
	Yes	2	38	50.0%
Proportion of hobbled camels (mean±SD)		0 (0% animals hobbled)-<br>2 (100% animals hobbled)	0.32±0.54	
Proportion of camels in shade (mean±SD)		0 (100% animals in shade)-<br>2 (0% animals in shade)	0.88±0.78	
**Total score Good housing: 0-18**				

[#] or not available

Table S4. Mean and standard deviation (SD) of scores of the measures collected at Herd level for the principles of Good health and Appropriate behaviour. The possible range for Total score of each principle is also reported.

Measure	Score	Mean	SD
Proportion of camels with a disease	0 (0% animals with a disease)- 2 (100% animals with a disease)	0.84	0.67
Proportion of camels with physical injuries	0 (0% animals with injuries)- 2 (100% animals with injuries)	0.12	0.29
Proportion of camels in pain	0 (0% animals in pain)- 2 (100% animals in pain)	0.07	0.26
Proportion of camels with injuries from halters or tethering	0 (0% animals with scars)- 2 (100% animals with scars)	0.22	0.41
Proportion of camels with cauterizations	0 (0% animals with cauterization)- 2 (100% animals with cauterization)	0.96	0.66
Proportion of camels with nose ring	0 (0% animals with nosering)- 2 (100% animals with nosering)	0.00	0.00
**Total score Good health: 0-12**			
Proportion of resting camels	0 (100% animals resting)- 2 (0% animals resting)	1.07	0.76
Proportion of standing quietly camels	0 (100% animals standing quietly)- 2 (0% animals standing quietly)	1.40	0.66
Proportion of aggressive camels	0 (0% animals aggressive)- 2 (100% animals aggressive)	0.14	0.31
Proportion of camels showing stereotypies	0 (0% animals showing stereotypies)- 2 (100% animals showing stereotypies)	0.00	0.00
Proportion of camels showing other abnormal behaviours	0 (0% animals in discomfort)- 2 (100% animals in discomfort)	0.22	0.40
**Total score Appropriate behaviour: 0-10**			

Table S5. Absolute (N) and relative (N%) frequency distribution of scores of the measures collected at Animal level for the principles of Good feeding and Good housing. The possible range for Total score of each principle is also reported.

Measure	Criteria	Score	N	N %
BCS	3 (good body condition)	0	30	22.7%
	2, 4 (moderate body condition)	1	77	58.3%
	0-1, 5 (lean or obese)	2	25	18.9%
Thirst Index	0	0	27	20.6%
	1	1	20	15.3%
	2-3	2	84	64.1%
**Total score Good feeding: 0 -4**				
Use of the shade	Yes	0	63	47.7%
	No	2	69	52.3%
Insects (presence)	No	0	112	84.8%
	Yes	2	20	15.2%
Tethering (presence)	No	0	127	96.2%
	Yes	2	5	3.8%
Hobbled (presence)	No	0	105	79.5%
	Yes	2	27	20.5%
Resting Behaviour	No	0	60	45.5%
	Yes	1	72	54.5%
**Total score Good housing: 0 - 10**				

BCS=Body Condition Score

Table S6. Absolute (N) and relative (N%) frequency distribution of scores of the measures collected at Animal level for the principles of Good health and Appropriate behaviour. The possible range for Total score of each principle is also reported.

**Measure**	**Criteria**	**Score**	**N**	**N %**
Disease (presence)	No	0	81	61.4%
	Yes	2	51	38.6%
Physical injuries (presence)	No	0	103	78.0%
	Yes	2	29	22.0%
Swollen joint (presence)	No	0	120	90.9%
	Yes	2	12	9.1%
Skin disorders (presence)	No	0	91	68.9%
	Yes	2	41	31.1%
Nasal and/or vulva discharge (presence)	No	0	123	93.2%
	Yes	2	9	6.8%
Gastro-enteric disorders (presence)	No	0	127	96.2%
	Yes	2	5	3.8%
Mastitis or abnormal udder (presence)	No	0	122	92.4%
	Yes	2	10	7.6%
Respiratory disorders (presence)	No	0	129	97.7%
	Yes	2	3	2.3%
Evident pain (presence)	No	0	122	92.4%
	Yes	2	10	7.6%
**Total score Good health**: **0 - 18**				
Positive social interactions	Yes	0	49	37.1%
	No	2	83	62.9%
Stereotypies	No	0	132	100.0%
	Yes	2	0	0.0%
Feeding	Yes	0	22	16.7%
	No	2	110	83.3%
Rumination	Yes	0	14	10.6%
	No	2	118	89.4%
Approaching Test (responses)	Positive	0	62	47.0%
	Neutral	1	41	31.1%
	Negative	2	29	22.0%
**Total score Appropriate behaviour: 0- 10**				

Figure S1. Partial indices (PIs) of the 4 welfare principles for Caretaker (**a**), Herd (**b**), and Animal level (**c**). Whiskers define the 5th and 95th percentile while the dots indicate the outliers (scores below the 5th percentile or above the 95th percentile).

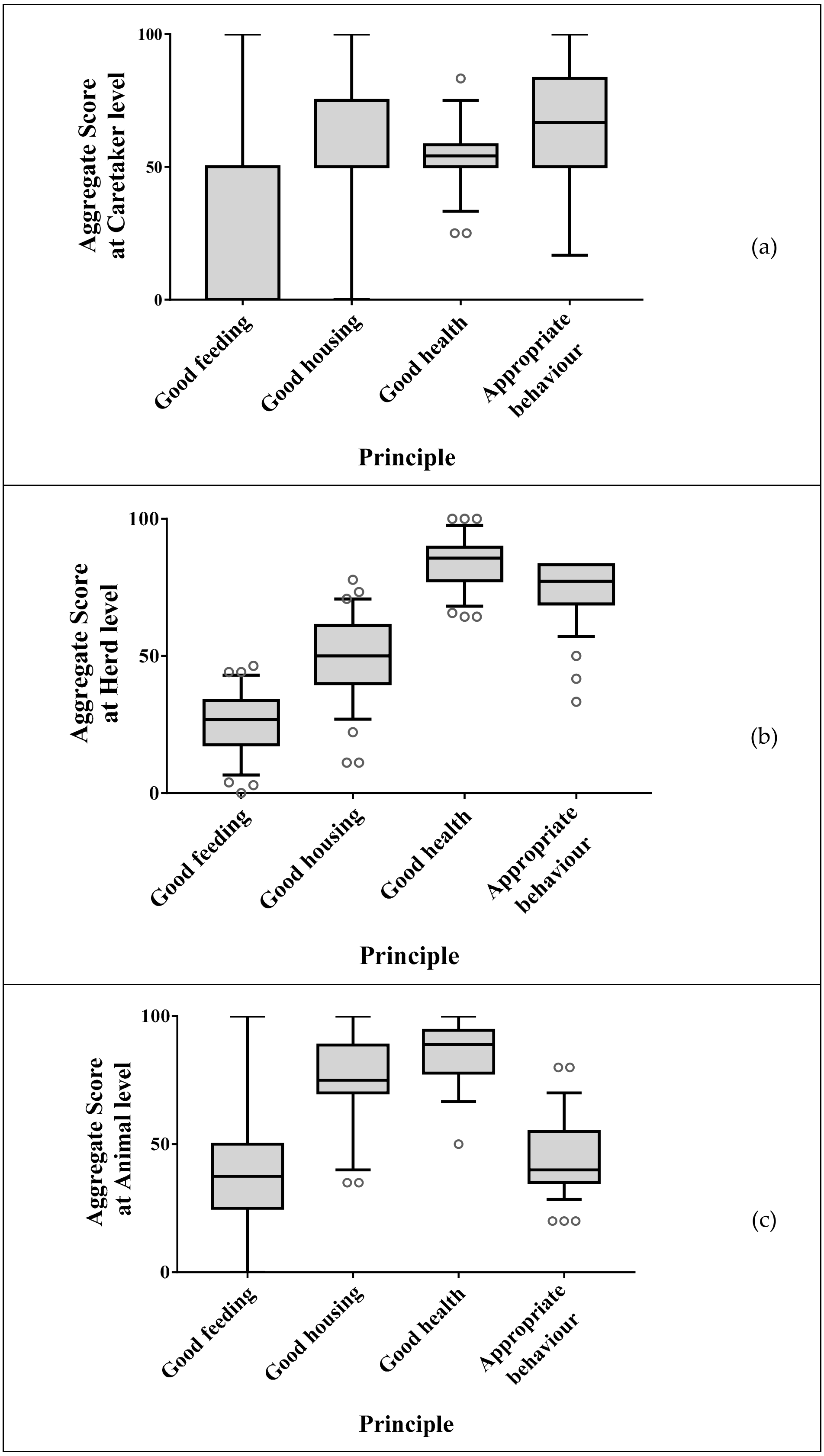

Figure S2. Classification of three pens (ID 34, ID 3, and ID 33) according to the indices aggregated at principle level and the bins of the Total welfare index. The class of each pen is circled in red for each classification system.

Pen ID	System of classification: Indices aggregated at Principle level	System of classification: Total welfare index
34	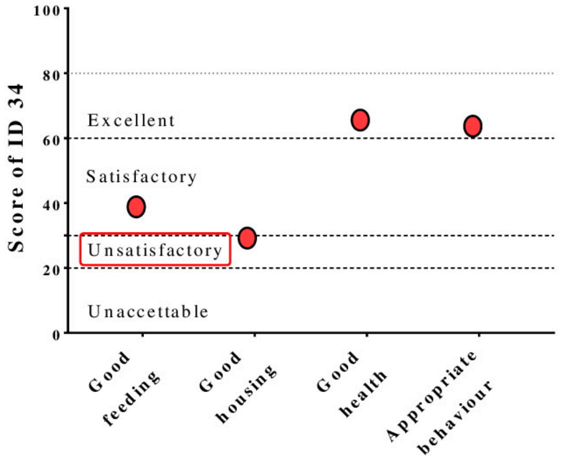	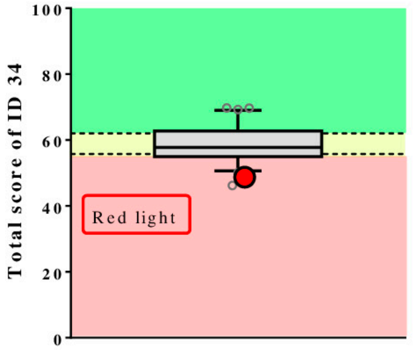
3	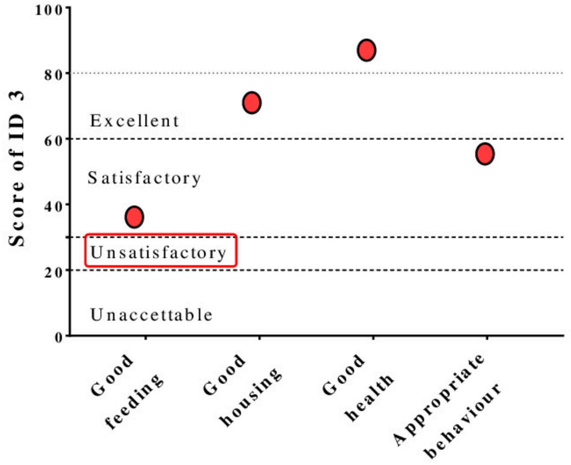	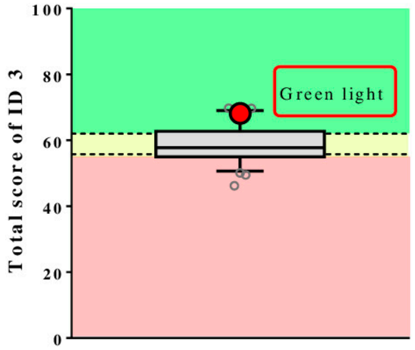
33	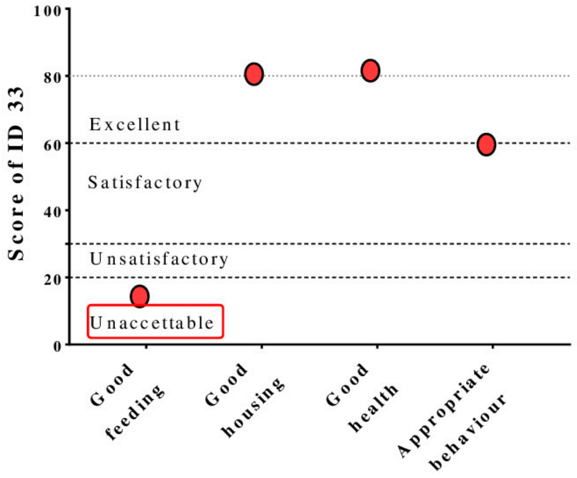	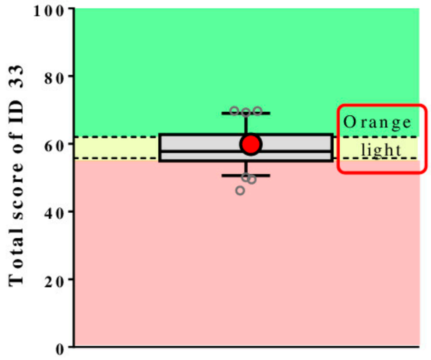
